# Supplementary material for: Five Inhibitory Receptors Display Distinct Vesicular Distributions in Murine T Cells
Source: Cells. 2023 Oct 31;12(21):2558. doi: 10.3390/cells12212558 (PMC10649679; doi:10.3390/cells12212558)
Supplement: Supplementary file 1 [file cells-12-02558-s001.zip › cells-2544383-supplementary/cells-2544383-supplementary.pdf]

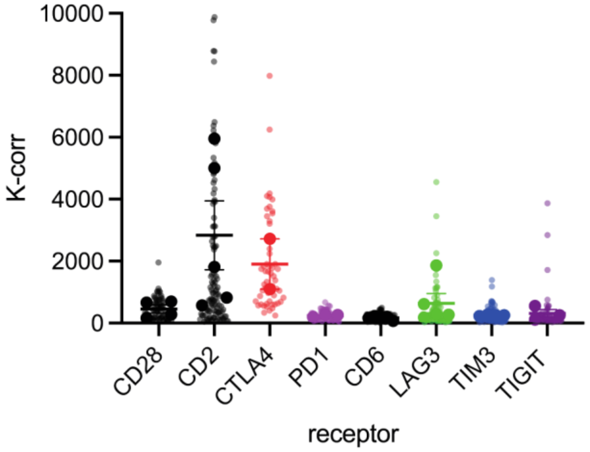

	CD28	CD2	CD6	CTLA-4	PD-1	LAG3	TIM3	TIGIT
CD28								
CD2	****		*				*	
CD6	****	****						
CTLA-4	****	****	****					
PD-1		****		****				
LAG3		****	***	****				
TIM3	**	****		****				
TIGIT	***	****		****	*			

Figure S1. The extent of subcellular clustering differs between the inhibitory receptors in CD4⁺ T cells
Ripley's k function k values for the same T cells as in Figure 1B. Small symbols are individual cells, large symbols are run averages. Statistical significance of differences between receptors is given in the table below, calculated based on individual cells on top in black, based on run averages on the left in grey.* $p<0.05$, ** $p<0.01$, *** $p<0.001$, **** $p<0.0001$; p values calculated using one-way ANOVA (run averages) and Kruskal-Wallis test (individual cells).

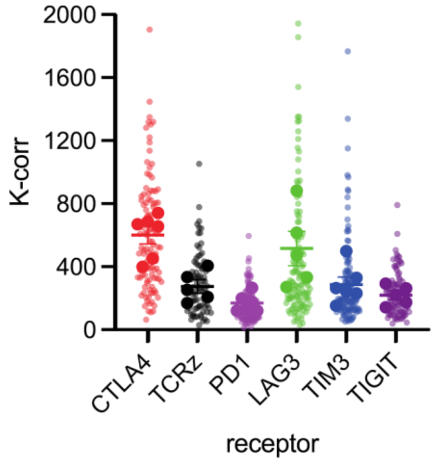

	TCRz	CTLA-4	PD-1	LAG3	TIM3	TIGIT
TCRz		**				
CLTA-4	****		****			
PD-1	****	****		**		
LAG3		***	****			*
TIM3		****	****	**		
TIGIT		***	**	****		

Figure S2. The extent of subcellular clustering differs between the inhibitory receptors in CD8^{+} T cells

Ripley's k function k values for the same T cells as in Figure 2B. Small symbols are individual cells, large symbols are run averages. Statistical significance of differences between receptors is given in the table below, calculated based on individual cells on top in black, based on run averages on the left in grey.* $p<0.05$, ** $p<0.01$, *** $p<0.001$, **** $p<0.0001$; p values calculated using one-way ANOVA (run averages) and Kruskal-Wallis test (individual cells).

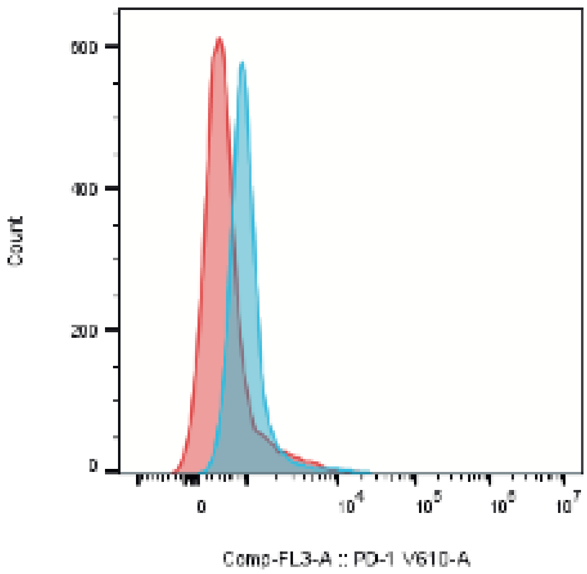

Figure S3. PD-1 is largely intracellular in T cells upon lack of TCR engagement
Representative flow cytometry data of $CD8^+$ T cells kept in IL-2 only without TCR engagement for 48h stained for PD-1 on the cell surface (red) or in the entire cell (blue).

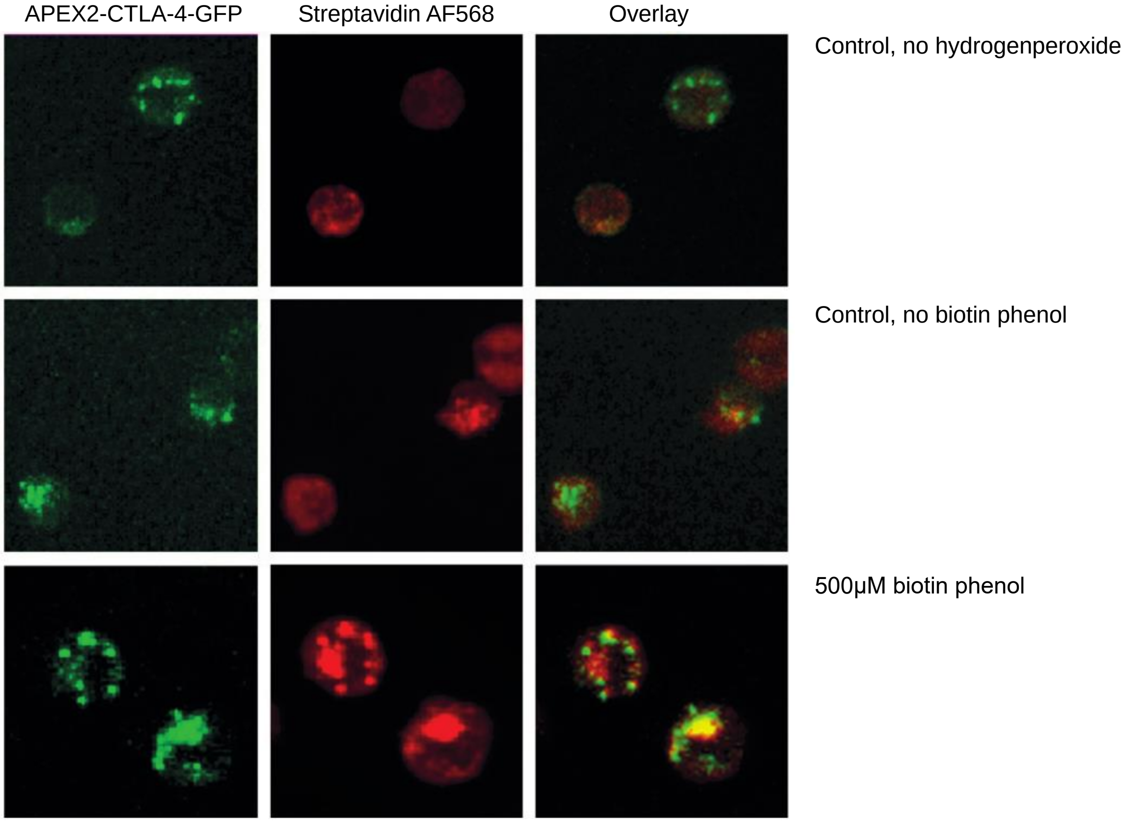

Figure S4. APEX2-mediated biotinylation is specific for vesicles expressing GFP-tagged inhibitory receptors
Representative data of CL4 T cells expressing APEX2-CTLA-4-GFP stained for biotinylated proteins with streptavidin AF648 after having undergone APEX2-mediated proximity biotinylation. GFP fluorescence in the left column, AF648 fluorescence in the middle. The top two rows are controls lacking H_2O_2 or biotin phenol.

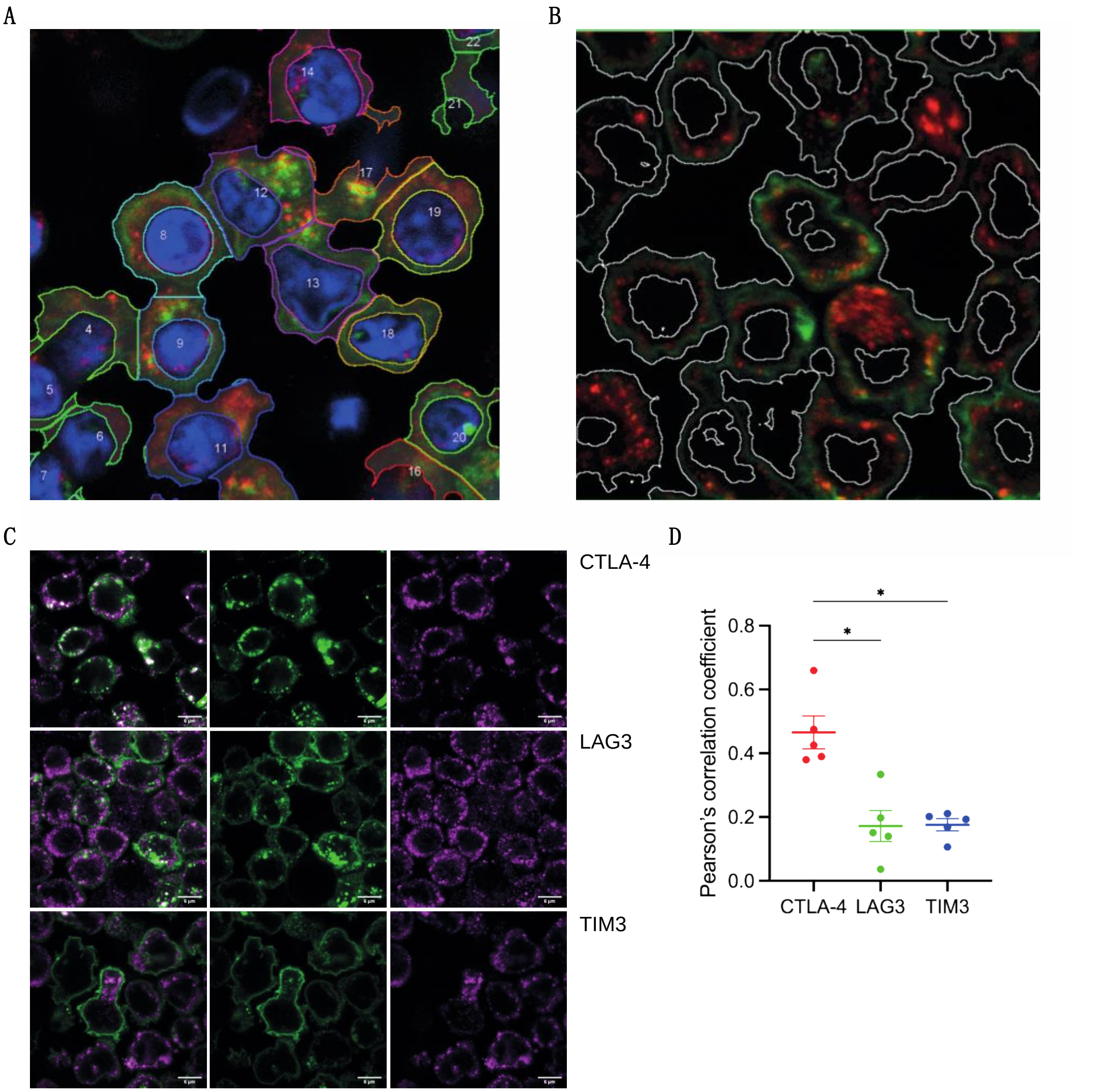

Figure S5. CTLA-4 is selectively enriched in endosomes late in T cell activation
A CL4 CTL expressing TIM3-GFP were activated by incubation with Renca target cells in the presence of 2μg/ml HA agonist peptide for 10min, fixed and stained with anti-EAA1 and for nuclei with Hoechst 33258. Given is a merged confocal z-stack midplane image of the anti-EAA1 (red), GFP (green) and Hoechst 33285 (blue) fluorescence with the cytoplasmic segmentation overlaid. Numbers indicate individual cells identified for subsequent measurement. **B** CL4 CTL expressing TIM3-GFP were activated by incubation with Renca target cells in the presence of 2μg/ml HA agonist peptide for 20min, fixed and stained with anti-EAA1. Given is a merged confocal z-stack midplane image of the anti-EAA1 (red) and GFP (green) fluorescence with the whole-field cytoplasmic segmentation overlaid. **C** CL4 CTL expressing GFP fusion proteins of the indicated inhibitory receptors were activated by incubation with Renca target cells in the presence of 2μg/ml HA agonist peptide for 20min, fixed and stained with anti-EAA1. Given are confocal z-stack midplane images of the EEA1 fluorescence (magenta), the GFP fluorescence (green) and merged images of all fluorescence data. **D** For the experiment in C, Pearson's correlation coefficients of the GFP and EAA1 fluorescence distributions across the segmented CTL cytoplasm of five entire imaging fields as mean±SEM. * $p<0.05$; p values calculated using one-way ANOVA

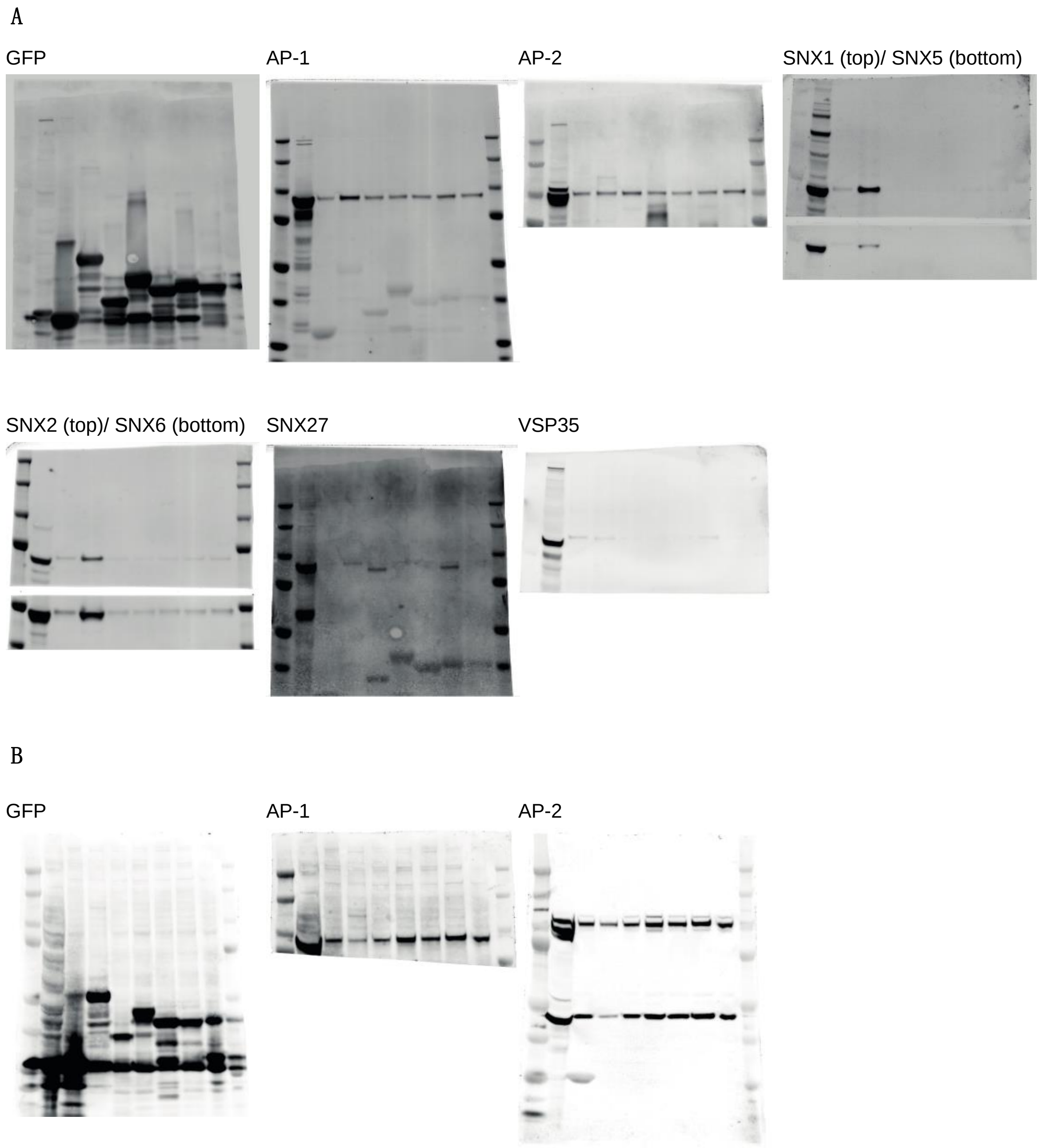

Figure S6. Full size Western blots for Figure 8

A, B Full size Western blots for Figure 8C (A) and 8G (B). Please note that we have commonly detected two proteins on the same Western blot, requiring us to cut the blot into two often unequal pieces.

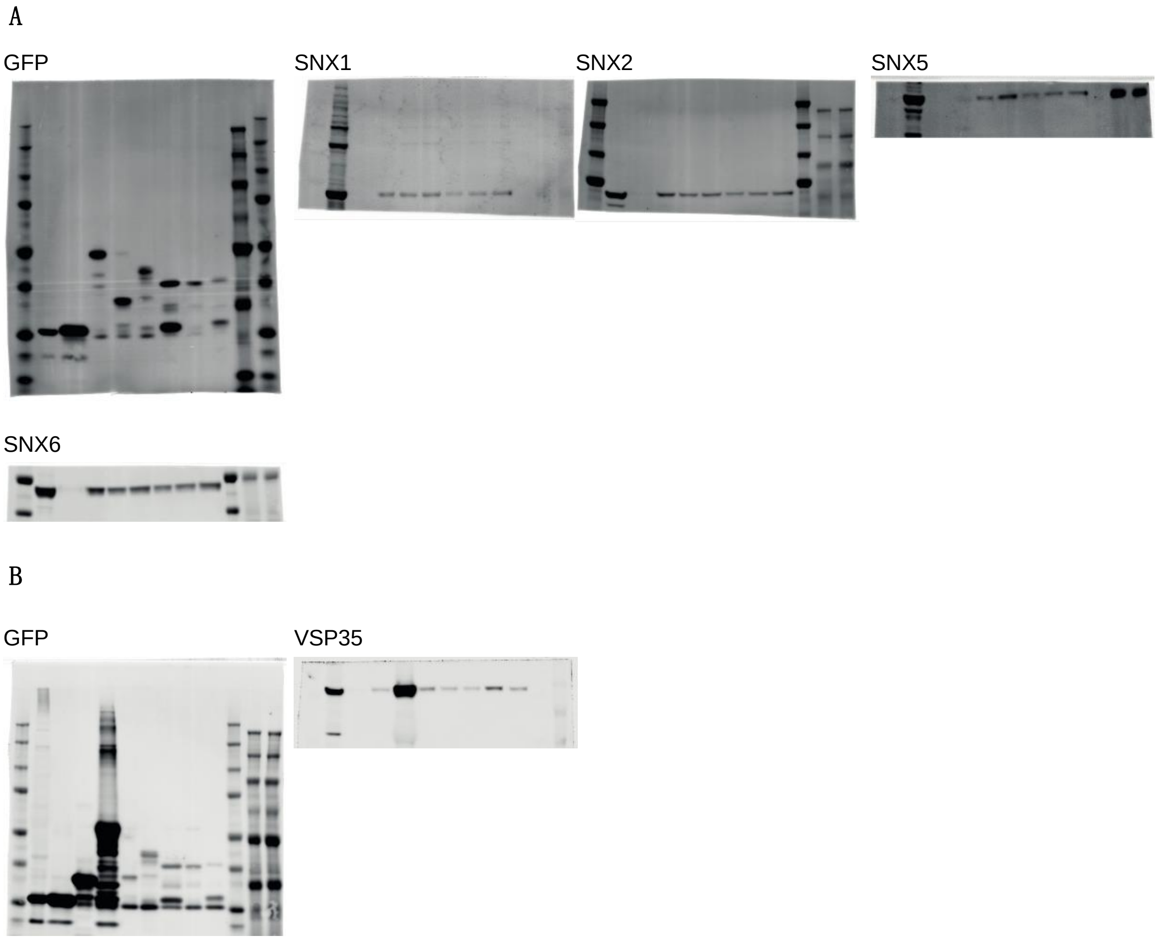

Figure S7. Full size Western blots for Figure 9

A, B Full size Western blots for Figure 9A (A) and 9F (B). Please note that we have commonly detected two proteins on the same Western blot, requiring us to cut the blot into two often unequal pieces.
