# Supplementary material for: Five Inhibitory Receptors Display Distinct Vesicular Distributions in Murine T Cells
Source: Cells. 2023 Oct 31;12(21):2558. doi: 10.3390/cells12212558 (PMC10649679; doi:10.3390/cells12212558)
Supplement: Supplementary file 1 [file cells-12-02558-s001.zip › cells-2544383-supplementary/File S1.zip!/MIA supporting information/MIA supporting information.pdf]

Installation and usage of MIA-based image analysis workflows.

ModularImageAnalysis (MIA) is an ImageJ plugin for creating modularised image and object analysis workflows. Full documentation for MIA is included at https://mianalysis.github.io. The following sections describe complete installation steps for the various MIA workflows described. In all instances, it is best to start with a new copy of Fiji and since Fiji instances can coexist on the same computer, shouldn't pose a problem.

- ***Determination of fluorescence at the cell edge versus interior and of distribution heterogeneity***

  Note:
  - Analysis incompatible with Apple Silicon Macs due to use of Tensorflow 1.15

  Installation:
  1. Download a new copy of Fiji from https://fiji.sc
  2. Launch Fiji and start the updater (Help > Update…) and add the update sites for the following:
     - CSBDeep
     - IJPB-plugins
     - ModularImageAnalysis (MIA)
     - StarDist

  Usage:
  1. Open Fiji and go to Plugins > ModularImageAnalysis (MIA) > MIA
  2. In MIA, click "Load" and select the provided "Determination of fluorescence at the cell edge workflow.mia" file. At this point, controls should be displayed.
  3. Select the DIC and GFP image files with the buttons to the right of "DIC file path" and "GFP file path", respectively.
  4. Select the provided "Determination of fluorescence at the cell edge model.zip" StarDist model file using the button to the right of "Model path (.zip)"
  5. Click "Run" to start the analysis. When complete, the progress bar at the bottom of MIA will go green. Results are stored in the same folder as the input DIC image file.

- ***Imaging of individual T cells to estimate association of inhibitory receptors with the cell edge***

  Note:
  - Analysis incompatible with Apple Silicon Macs due to use of Tensorflow 1.15

  Installation:
  1. Download a new copy of Fiji from https://fiji.sc
  2. Launch Fiji and start the updater (Help > Update…) and add the update sites for the following:
     - IJPB-plugins
     - ModularImageAnalysis (MIA)
  3. Download the DeepImageJ version 2.1.16 jar file from https://github.com/deepimagej/deepimagej-plugin/releases/download/2.1.16/DeepImageJ_-2.1.16.jar and place it in the "plugins" folder of the new copy of Fiji
  4. Download and extract the DeepImageJ dependencies (version 2.1.16) folder from https://github.com/deepimagej/deepimagej-plugin/releases/download/2.1.16/dependencies_2116.zip and place the contents in the "jars" folder of the new copy of Fiji.
  5. Extract the supplied "Imaging of individual T cells model.zip" folder to the "models" folder of the new copy of Fiji (it may be necessary to create this folder).

  Usage:
  1. Open Fiji and go to Plugins > ModularImageAnalysis (MIA) > MIA
  2. In MIA, click "Load" and select the provided "Imaging of individual T cells workflow.mia" file. At this point, controls should be displayed.
  3. Select the image file to process by clicking the button to the right of "Input path".
  4. Click "Run" to start the analysis. When complete, the progress bar at the bottom of MIA will go green. Results are stored in the same folder as the input image file.

- ***Colocalization analysis in dual color TIRF experiments***

  Installation:

  1. Download a new copy of Fiji from https://fiji.sc
  2. Launch Fiji and start the updater (Help > Update…) and add the update sites for the following:
     - IJPB-plugins
     - ModularImageAnalysis (MIA)

  Usage:

  1. Open Fiji and go to Plugins > ModularImageAnalysis (MIA) > MIA
  2. In MIA, click “Load” and select the provided “Colocalization analysis in dual color TIRF.mia” file. At this point, controls should be displayed.
  3. Select the image file to process by clicking the button to the right of “Input path”.
  4. Click “Run” to start the analysis. When complete, the progress bar at the bottom of MIA will go green. Results are stored in the same folder as the input image file.

- ***Membrane colocalization in 2D***

Installation:

1. Download a copy of Fiji version 20201104-1356 from https://downloads.imagej.net/fiji/archive/20201104-1356/
   - Note: Please do not run the updater on this version as we want to retain Fiji in its current state
2. Download the following jar files and put them in the "plugins" folder of the new copy of Fiji:
   - MIA version 0.18.2 from https://github.com/mianalysis/mia/releases/download/v0.18.2/MIA_-0.18.2.jar
   - MorphoLibJ version 1.4.1 from https://github.com/ijpb/MorphoLibJ/releases/download/v1.4.1/MorphoLibJ_-1.4.1.jar
   - RidgeDetection version 1.4.0 from https://github.com/thorstenwagner/ij-ridgedetection/releases/download/v1.4.0/ij_ridge_detect-1.4.0.jar

Usage:

1. Open Fiji and go to Plugins > Bristol WBIF > MIA (Modular Image Analysis)
2. In MIA, click "Load" and select the provided "Membrane colocalization in 2D workflow.mia" file. At this point, controls should be displayed.
3. Select the image file to process by clicking the button to the right of "Input path".
4. Select the provided Weka Trainable Segmentation model by clicking the button to the right of "Classifier file path".
5. Click "Run" to start the analysis. When complete, the progress bar at the bottom of MIA will go green. Results are stored in the same folder as the input image file.

- ***Membrane colocalization in 3D***

Installation:

1. Download a copy of Fiji version 20201104-1356 from https://downloads.imagej.net/fiji/archive/20201104-1356/
    - Note: Please do not run the updater on this version as we want to retain Fiji in its current state
2. Download the following jar files and put them in the "plugins" folder of the new copy of Fiji:
    - MIA version 0.18.15 from https://github.com/mianalysis/mia/releases/download/v0.18.2/MIA_-0.18.2.jar
    - MIA_MATLAB version 1.2.3 from https://github.com/mianalysis/mia-matlab/releases/download/v1.2.3/MIA_MATLAB-2018b-1.2.3.jar
    - MorphoLibJ version 1.4.1 from https://github.com/ijpb/MorphoLibJ/releases/download/v1.4.1/MorphoLibJ_-1.4.1.jar
    - RidgeDetection version 1.4.0 from https://github.com/thorstenwagner/ij-ridgedetection/releases/download/v1.4.0/ij_ridge_detect-1.4.0.jar
3. Download and install the MATLAB Compiler Runtime version 9.5 (2018b) from https://ssd.mathworks.com/supportfiles/downloads/R2018b/deployment_files/R2018b/installers/win64/MCR_R2018b_win64_installer.exe
    - Note: This step can be skipped if a full copy of MATLAB 2018b exists on the system
4. From the new MATLAB Compiler Runtime installation, copy the "javabuilder" file to the "jars" folder of the new copy of Fiji.
    - Note: From the root MATLAB folder this can be found at "MATLAB Runtime\v95\toolbox\javabuilder\jar\javabuilder.jar"

Usage:

1. Open Fiji and go to Plugins > Bristol WBIF > MIA (Modular Image Analysis)
2. In MIA, click "Load" and select the provided "Membrane colocalization in 3D workflow.mia" file. At this point, controls should be displayed.
3. Select the image file to process by clicking the button to the right of "Input path".
4. Click "Run" to start the analysis. When complete, the progress bar at the bottom of MIA will go green. Results are stored in the same folder as the input image file.
